# Effect of repeated exposure to an identical multi-user virtual reality anaphylaxis scenario on paramedic trainee performance: a randomized controlled trial

**DOI:** 10.1038/s41598-026-69133-x

**Published:** 2026-09-03

**Authors:** Julian Giese, Jan P. Ehlers, Olaf Weichert, Julia Nitsche

**Affiliations:** 1 Witten/Herdecke University, Chair of Didactics and Educational Research in Healthcare, Alfred-Herrhausen-Straße 50, 58455 Witten, North Rhine-Westphalia Germany; 2 Simulation and Emergency Academy, Helios Hospital, Lutherplatz 40, 47805 Krefeld, North Rhine-Westphalia Germany

**Keywords:** Virtual Reality, Paramedic Education, Simulation-Based Training, Repeated Exposure, Scenario-Specific Performance, Anaphylaxis Management, Health care, Medical research

## Abstract

Immersive virtual reality (VR) may provide reproducible environments for training time-critical emergency workflows, but evidence in paramedic education remains limited. In this single-center randomized controlled trial, 125 German paramedic trainees participated in dyadic simulations after allocation to either one standardized multi-user VR anaphylaxis scenario (VR-C, n = 61) or repeated exposure to the identical scenario (VR-I, n = 64). The common assessment scenario was VR2. Scenario performance was assessed jointly for each dyad using a guideline-derived 62-item checklist and separate time metrics for correct working diagnosis and first intramuscular (IM) epinephrine administration. In VR2, the VR-I group achieved higher total scores than the VR-C group (42.6 vs. 31.1 of 62 points; mean difference 11.5, 95% CI, 9.8 to 13.3) and reached the working diagnosis (1.45 vs. 2.90 min) and first IM epinephrine administration (4.58 vs. 7.43 min) earlier. Knowledge test scores did not show corresponding between-group differences. VR-related symptoms were generally mild, and no simulation was discontinued. Repeated exposure to an identical VR anaphylaxis scenario was associated with improved subsequent scenario-specific, guideline-based performance within the VR environment. The findings support VR as a reproducible tool for deliberate rehearsal, but do not establish transfer to novel cases, real-world care, or superiority over conventional simulation. **Trial registration:** This trial was retrospectively registered on the Open Science Framework (OSF) prior to journal submission. Data collection was completed at the time of registration. The registration includes the analysis plan and outcome definitions used for this manuscript. Analyses not specified in the registration are labeled exploratory. Registered at the OSF, registration DOI: 10.17605/OSF.IO/V8XME, registration date: 6 February 2026.

## Introduction

For emergency medical service (EMS) trainees, opportunities to acquire and consolidate practical competencies under realistic conditions are inherently limited. Real-life emergency missions are characterized by substantial psychological stress, time pressure, and a very low tolerance for mistakes. Moreover, increasing clinical complexity and the low prevalence of certain high-acuity conditions restrict trainees’ exposure to critical scenarios during routine training^1,2^.

To address these challenges, paramedic education programs widely rely on simulation-based training using actors and mannequins^3^. Despite their proven educational value, traditional simulation approaches in paramedic education face inherent limitations in standardization and scalability, particularly when aiming to recreate dynamic, high-stress emergency environments and diverse clinical conditions^4^.

As a result, these clinically relevant aspects of emergency care may be only partially accessible within traditional training formats. Immersive virtual reality (VR) environments offer the potential to complement existing educational strategies by providing simulations that approximate operational stressors while maintaining a safe learning environment^5^.

In VR technology, simulation software and VR head-mounted displays (HMDs) that adjust their images to the movements of the users create virtual spaces that feel close to reality. The goal is to elicit a sense of being physically present in a nonphysical environment such that the VR setting is perceived as real by the user. This subjective experience is commonly referred to as immersion^6^.

This technology has already been used successfully in various medical fields. With the help of VR, surgical procedures can be practiced^7^, physiological processes can be illustrated^8^, and anatomy can be studied in a safe and ethical way^9^. International evidence suggests that, compared with traditional teaching methods, VR-based interventions in healthcare professions lead to superior knowledge acquisition and skill development^10^.

This potential is also increasingly recognized in emergency medical training^11–15^. VR enables visual representations that surpass the limitations of traditional methods^16^. Moreover, VR simulations can be easily reproduced, as training scenarios are digitally predefined, stored, and repeatedly loaded in an identical form. This enables standardized training and assessment while simultaneously reducing staffing requirements and associated costs^5^.

However, haptic procedural skills, such as establishing vascular access or performing hands-on airway maneuvers, cannot be adequately practiced within a purely virtual environment^17,18^. VR simulations can therefore serve only as a supplement to traditional simulations, not as a replacement.

Some VR systems for emergency medical services training have already been developed^14,16,19^, such as the software i:medtasim (TriCAT GmbH, Germany), which was used in this study. A study by Lerner et al.^16^ previously employed this software and demonstrated learning effectiveness within a small sample (n = 18), offering preliminary evidence that warrants further investigation in larger cohorts. Some published systematic reviews in other medical fields focus on different educational dimensions, including learning outcomes, usability, and learner experience, particularly in the domains of anatomy education and the acquisition of surgical skills^7,9,20,21^. For paramedic training, however, the current evidence base is limited and fragmented, and empirical evidence for VR-based training remains scarce^16,22,23^. The use of VR in paramedic education remains at an early stage, with limited evidence on how established teaching methods can be effectively transferred and applied within VR-based learning environments^6^. In Germany, a considerable amount of data and experiences have been published in specialized EMS journals but not in any international context^24–27^.

A key consideration in the implementation of VR-based training is the occurrence of VRISE (virtual reality-induced symptoms and effects^28^), which encompass nausea, dizziness, disorientation, headaches, fatigue, and other transient adverse effects^28–30^. VRISE comprise a spectrum of adverse responses commonly referred to as motion sickness, cybersickness, and simulator sickness – terms that are frequently used interchangeably but differ in their underlying causes^31^. A common cause of VRISE is sensory conflict, particularly when visually perceived self-motion in a virtual environment is not matched by corresponding physical or vestibular cues or when limitations of the simulation itself induce perceptual inconsistencies^31^. The likelihood and severity of VRISE depend on the VR system, scenario design, and participant characteristics. The HMDs, such as the HTC VIVE Focus 3 (HTC Corporation, Taiwan) used in this study, introduce sensory and motor inputs that may elicit VRISE^29,30^. Furthermore, the software uses controller-based teleportation, which has been associated with the occurrence of VRISE symptoms^16^.

Although physiological parameters such as heart and respiratory rates, gastric myoelectrical activity, and electrodermal activity may provide objective markers of VRISE^32^, the present study focused on subjective symptom reporting, which constitutes the most directly relevant outcome in applied educational VR settings.

Age may play a role in individual susceptibility to VRISE, but the findings are inconsistent^30^. Gender does not appear to be a robust predictor^30^. Longer VR exposure durations are generally associated with greater symptom severity, whereas repeated exposures may attenuate VRISE through habituation effects^29^. Assessing VRISE is crucial not only for participant safety but also for optimizing VR-based medical training and guiding future research on mitigating adverse effects through both system design and pre-exposure risk stratification^30,32^.

Anaphylaxis was selected as the study scenario because it combines visually salient clinical signs, including urticaria and upper-airway swelling, with a standardized, time-critical treatment pathway in which rapid recognition and prompt intramuscular epinephrine administration are central to guideline-concordant care^36,37,47^.

The primary research question of this study was whether repeated exposure to the same anaphylaxis scenario leads to measurable improvements in paramedic trainee performance during a subsequent exposure to the same virtual emergency scenario. Training effectiveness was therefore conceptualized as scenario-specific performance in time-critical, guideline-based decision-making and intervention within a reproducible and standardized VR environment. The study was not designed to test transfer to different emergency scenarios, conventional simulation settings, or real-life paramedic performance.

The secondary objectives were to examine the safety and tolerability of immersive VR training by assessing VRISE and to explore whether individual participant characteristics modulate performance outcomes or symptom burden. In addition, the study sought to evaluate the coherence between objectively measured performance in VR simulations and independently assessed medical knowledge.

## Methods

### Study design

This single-center randomized controlled trial is reported in accordance with the CONSORT 2025 guidelines^33^ and was conducted between April and August 2024 at the Simulation and Emergency Academy (SiNA), Helios Hospital Krefeld, Germany. The study employed a parallel-group randomized design. Participants allocated to the intervention group (VR-I) completed the same standardized VR anaphylaxis scenario twice, with VR1 serving as the additional exposure and VR2 serving as the common assessment scenario. Participants allocated to the control group (VR-C) completed only VR2 and therefore encountered the anaphylaxis scenario for the first time during the common assessment. Accordingly, the trial evaluated the effect of repeated exposure to an identical VR-based emergency scenario, rather than transfer between different clinical scenarios.

**Fig. 1:**
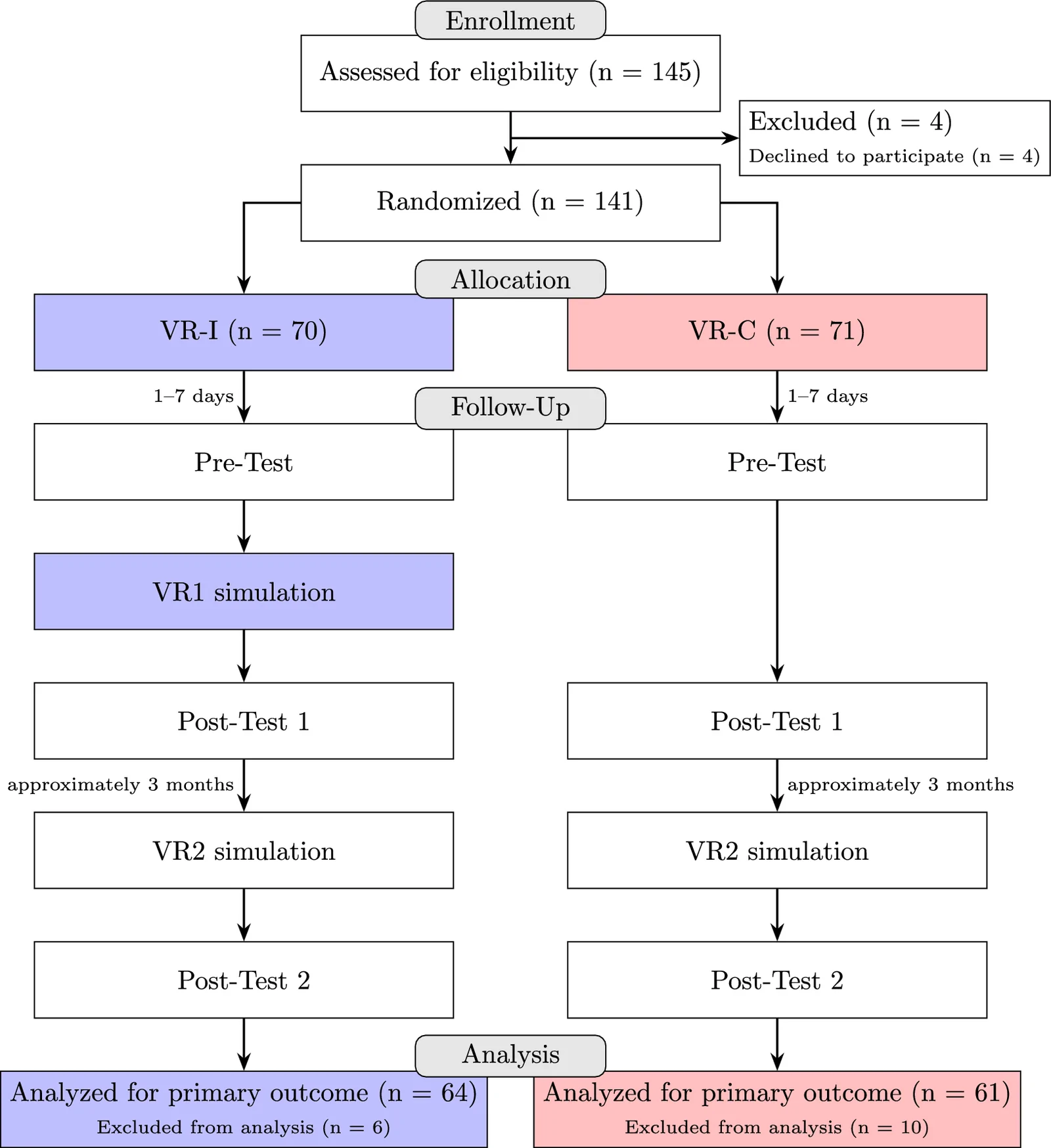
Flow diagram of the progress through the phases of this study.

Preparatory work began in February 2024, with standardized familiarization sessions conducted for all enrolled training cohorts. A predefined familiarization workflow was developed to ensure a consistent introduction to the VR environment across cohorts. Each session started with a classroom-based introduction and demonstration of the VR technology, including the hardware, software interface, and controllers, delivered to the entire training class. This was followed by individual hands-on familiarization, during which each participant completed a short practice scenario in the VR environment to become accustomed to navigation, interaction, and basic system handling. An overview of this study program is shown in Fig. 1. There was no public or patient involvement in the design, conduct, or reporting of the trial.

The i:medtasim platform and the institutional VR setup were novel to all participants before the standardized familiarization, as the present study represented the first use of VR simulation at SiNA. Participants did not have access to or use i:medtasim outside the scheduled study sessions, and no additional VR simulations were conducted at SiNA during the study period.

No study-provided debriefing, corrective instruction, or additional anaphylaxis simulation was delivered between VR1 and VR2. Routine curricular teaching and real-world clinical exposure outside the study were not systematically recorded.

### Participants

In Germany, paramedics (Notfallsanitäter, the German professional paramedic qualification) complete a three-year vocational training program without a university degree^34^.

The participants were full-time EMS trainees enrolled in either their first or second year of the three-year vocational paramedic training program at SiNA. The eligibility criteria required participants to be at least 18 years of age, provide written informed consent, and feel physically capable of participating in VR simulations. Before enrollment and randomization, all training cohorts received a standardized familiarization session with the VR system. Trainees who did not feel physically able to participate or who declined participation because of previous VR-related discomfort were not randomized. This preselection was implemented for participant safety and feasibility but is relevant when interpreting the tolerability outcomes. No external recruitment occurred, and no direct compensation was given, as all eligible trainees from the enrolled cohorts were invited to participate during their paid training time.

Using Cohen’s approach to statistical power analysis^35^, an a priori sample-size calculation indicated that 63 participants per group were required to detect a medium effect size ($\eta ^2$ = 0.06) with 80% power at *α = 0.05* using one-way ANOVA. Accounting for an anticipated dropout rate of approximately 10%, eight training cohorts (four from each of the two academic years) were enrolled, resulting in 145 trainees assessed for eligibility. The participants were individually allocated using a simple randomization procedure within cohorts (dice rolls), aiming for approximately balanced group sizes while accommodating the academic schedule. To minimize selection bias, the allocation sequence was generated and implemented immediately prior to participation, and group assignment was not disclosed to participants until the start of the VR simulation.

Owing to the nature of the intervention, the blinding of participants and study personnel was not feasible. The participants were aware of their group assignment, and the study coordinator, who conducted randomization, also supervised all the simulations and completed real-time performance assessments. To minimize potential bias, all assessments used a standardized scenario-specific performance assessment checklist with objective criteria. Participant questionnaire responses were recorded under participant-generated four-character pseudonym codes, which were used solely to link questionnaire data with performance assessments across time points.

Predefined stopping criteria at the individual participant level included withdrawal of informed consent at any time. Potential reasons for withdrawal included VRISE that precluded continued participation. The participants were also free to withdraw consent without providing a reason and with immediate effect. No interim analyses or trial-level stopping guidelines were defined.

### VR technology and scenario

**Fig. 2:**
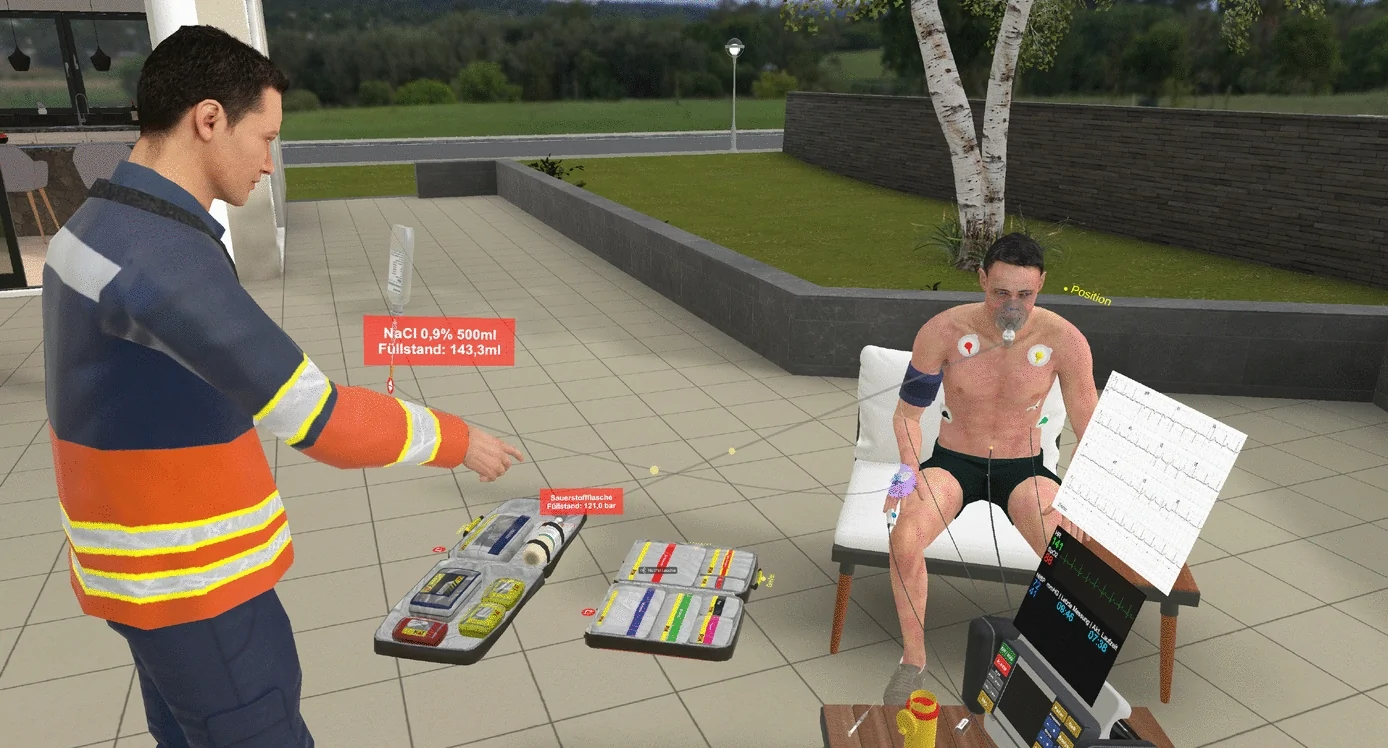
Screenshot from the virtual emergency scenario displayed from the first participant’s first-person perspective. The visible person depicts the second participant in the shared VR environment. Unaltered screenshot from i:medtasim (TriCAT GmbH, Germany), reproduced with permission.

All simulations used HTC VIVE Focus 3 HMDs with wireless controllers and the EMS-focused multi-user simulation software i:medtasim (see Fig. 2). Each simulation was completed by a dyad comprising two consented study participants who had both been individually randomized to the same study group. Dyads were formed within training cohorts according to participant availability and operational scheduling. No fixed team leader or assistant role was assigned, and both participants were free to communicate, make decisions, and perform actions throughout the scenario. For repeated exposure in the VR-I group, the same dyad composition was retained whenever operationally feasible, although pairings could change between VR1 and VR2.

All VR simulations presented the same standardized scenario of grade III anaphylaxis with anaphylactic shock and upper airway involvement^36^. VR1 and VR2 were identical with respect to dispatch keyword, environment, patient presentation, symptom pattern, clinical trajectory, available equipment, expected diagnostic and therapeutic actions, and scoring criteria. A standardized workflow was used to ensure procedural consistency and reproducibility across all simulation runs. The complete workflow is provided in the Supplementary Information. Participant interventions dynamically influenced the virtual patient’s physiological parameters, which were adapted in real time by the study instructor to reflect the consequences of the actions taken. However, the underlying scenario structure and expected management pathway remained unchanged across exposures.

**Fig. 3:**
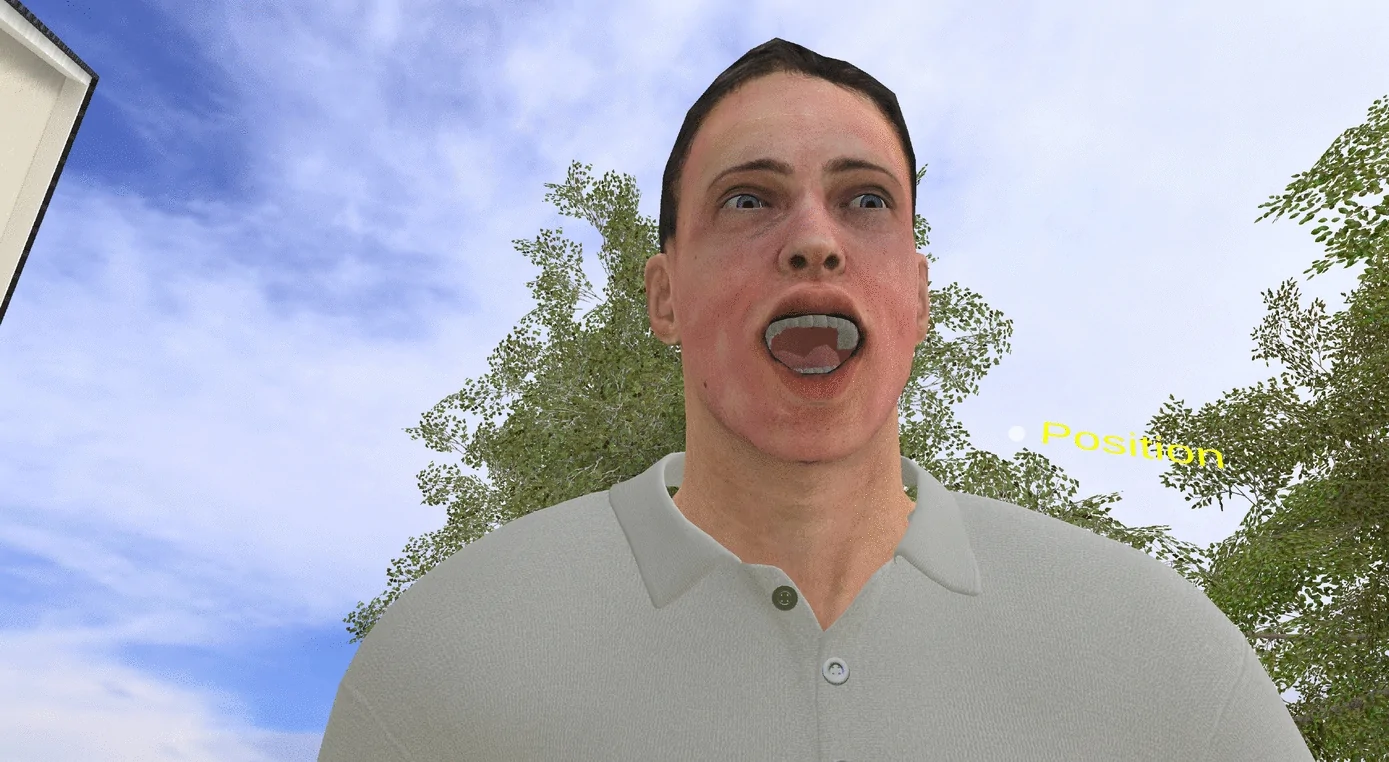
Screenshot from the VR simulation showing the virtual patient with urticaria from the first-person perspective after opening the patient’s mouth, illustrating upper airway involvement with visible uvular swelling consistent with anaphylaxis. Unaltered screenshot from i:medtasim (TriCAT GmbH, Germany), reproduced with permission.

Within the virtual scenario, the patient displayed typical manifestations of anaphylaxis, such as generalized urticaria and uvular swelling, as depicted in Fig. 3.

Throughout the simulations, the participants were able to interact both with one another and with the supervising instructor. The instructor additionally provided the voice of the virtual patient, facilitating direct communication between the participants and the patient. The participants could access virtually represented devices and medications and use them in virtual form. This included, among others, backpacks containing emergency equipment for airway management, emergency medications, a full ECG monitoring system with defibrillation capability, suction devices, an oxygen supply, and other standard emergency medical equipment (see Fig. 2). To ensure that the participants could not collide with each other while moving in the virtual space, half of the accessible physical space was allocated to each of them.

Scenario performance was assessed as the joint performance of the dyad. For each simulation run, the study coordinator completed one scenario-specific checklist and recorded one set of time-based outcomes for the team. These shared performance values were subsequently linked to both members of the dyad for integration with their individually completed questionnaire data.

### Development and scoring of the scenario-specific checklist

The checklist was developed specifically for this study to operationalize best-practice management of grade III anaphylaxis in the German paramedic training context. It was designed as a transparent, checklist-based assessment of guideline-concordant actions rather than as a psychometrically validated competence assessment instrument. Its content was derived from established emergency medical assessment frameworks and clinical guidance, including the scene, safety, support, and situation framework (SSSS) for structured situational anticipation, the initial global patient impression (“first look”), the exsanguination, airway, breathing, circulation, disability, and exposure approaches with catastrophic hemorrhage control (xABCDE) for the systematic identification of life-threatening conditions, the mnemonic for symptoms, allergies, medications, past medical history, last oral intake, events leading to the incident, and risk factors (SAMPLER) for focused collection of medical history, national and international anaphylaxis recommendations, and the German paramedic treatment pathway for anaphylaxis (BPR; Behandlungspfad Rettungsdienst)^36,39–45,47^.

Before implementation, the draft checklist underwent an informal expert content review by two physicians with prehospital emergency medicine qualifications. The review focused on medical accuracy, completeness, alignment with guideline-based treatment algorithms and operational EMS practice, and the clarity of the individual criteria. The checklist was finalized following this review. This review did not constitute a formal content-validity study or psychometric validation.

The final instrument comprised 62 dichotomously scored performance criteria distributed across five domains. Each item was scored as fulfilled (1 point) or not fulfilled (0 points), resulting in a maximum total score of 62 points. Although all individual items were weighted equally at the scoring level, clinically central actions were intentionally represented by several separate criteria. For example, IM epinephrine administration was captured not only as performance of the intervention itself but also through separate criteria for correct medication, dose, form of application, and injection site. This structure was intended to reflect the clinical importance of time-critical anaphylaxis management while preserving transparent item-level scoring.

Time to correct working diagnosis, time to first IM epinephrine administration, and total duration were recorded separately and were not mathematically included in the total checklist score. Nevertheless, because several checklist domains reflected procedural sequencing and time-critical priorities, faster recognition of the clinical pattern and greater familiarity with the scenario workflow may have indirectly facilitated higher checklist performance.

The checklist did not undergo formal psychometric validation or formal pilot testing before the trial. In addition, no inter-rater reliability metrics were calculated because real-time performance assessment was conducted by a single study coordinator using standardized criteria. These methodological constraints are considered when interpreting the performance outcomes.

The study coordinator supervised all VR sessions, provided standardized scenario initiation, and monitored technical execution.

### Outcomes and questionnaires

The primary outcome was performance in the standardized VR2 assessment scenario, operationalized by the total score of the scenario-specific checklist and the time to correct working diagnosis and first IM epinephrine administration. These time-based metrics were selected because early recognition of anaphylaxis and timely epinephrine administration represent central priorities in guideline-based emergency care^38,46^.

For all time-based outcome measures, the start and end points of each VR simulation were predefined. The time measurement commenced once both participants were wearing HMDs, held the controllers, the scenario had been initiated, the standardized scenario introduction had been delivered, and the participants were explicitly instructed to begin the scenario. A VR simulation was considered complete when one of the following criteria was met: (1) all predefined items of the standardized checklist had been fulfilled; (2) at least one participant requested termination of the simulation; (3) the maximum simulation duration of 25 minutes was reached; (4) no further clinically relevant actions or ideas were introduced by the participants despite prolonged observation; or (5) one or both participants explicitly stated that all necessary on-scene measures had been completed and that patient transport would be initiated.

All other outcomes, including the occurrence of VRISE and their association with individual participant characteristics, were considered secondary and provided additional context.

All questionnaires were administered digitally via the LimeSurvey platform using either participant-owned smartphones or study-provided tablets, employing the institutional LimeSurvey campus license of Witten/Herdecke University. No time constraints were imposed. The participants were instructed to answer on the basis of their current knowledge without consulting external sources or discussing with peers. This procedure was intended to standardize testing conditions for the knowledge items while allowing self-report items to capture participants’ current perceptions. The questionnaires were completed at three time points: pre-test (prior to any VR exposure), post-test 1 (immediately after VR1 for the VR-I group or at the corresponding time point for the VR-C group), and post-test 2 (immediately after VR2 for both groups). Each assessment included a distinct set of questions (see Table 1). Over the course of the study, the following data were collected from the questionnaires:

**Sociodemographic and training-related data:** A custom-designed questionnaire collected baseline participant characteristics, including age, gender, highest educational qualification, prior vocational training (particularly in emergency medical services) and years of EMS experience. Additionally, information on prior experience with VR technology and whether the subjects wore eyeglasses was collected.

**Theoretical knowledge on anaphylaxis treatment:** Clinical knowledge related to anaphylaxis management was assessed using 14 custom-developed questions covering pathophysiology, treatment principles, medication dosing, differential diagnosis, and clinical assessment algorithms. The content was based on the current German S2k clinical guidelines for acute treatment and management of anaphylaxis^47^ and the standard operating procedures of the German EMS^44,45^.

**Questions about VR-related experiences:** A custom 18-item questionnaire assessed participants’ perceptions of the VR simulation, including immersion and realism, technical quality and usability, perceived learning outcomes, attitudes toward VR integration, and perceived VRISE. All items were rated on a five-point Likert-type response scale coded as 0 (*strongly disagree*), 1 (*somewhat disagree*), 2 (*partly agree/partly disagree*), 3 (*somewhat agree*), and 4 (*strongly agree*). Higher scores indicated stronger agreement with the respective statement; for the two VRISE items, higher scores therefore indicated greater symptom burden. Several items were adapted from previous research using the same VR software platform^16^.

**Table 1:** Questionnaire components administered at each assessment time point.

Contents	Pre-Test	Post-Test 1	Post-Test 2
Sociodemographic data	*√*	—	—
Education-related data	*√*	—	—
Questions about VR simulation and experience$^1$	—	*√*	*√*
Questions on guideline-based anaphylaxis management	*√*	*√*	*√*

$^1$Administered at post-test 1 only in the VR-I group.

In the 18-item VR questionnaire, conceptually related items were grouped into five multi-item subscales and one single-item measure reflecting key dimensions of the VR simulation experience. Immersion comprised items 1, 2, 3, 13, and 15; competency progression comprised items 4, 5, 16, and 17; usability comprised items 9, 11, 12, and 14; attitudes toward VR integration comprised items 6 and 10; VRISE comprised items 7 and 8; and presimulation topic awareness was represented by item 18. Multi-item subscale scores were calculated as the mean of their constituent items; presimulation topic awareness was analyzed as a single item.

All questionnaires and assessment instruments used in this study are provided in the Supplementary Information.

### Statistical analysis

The data were exported from LimeSurvey to Microsoft Excel versions 2406–2409 (Microsoft Corporation, Redmond, WA, USA) for data cleaning and preliminary descriptive analyses. Statistical analyses were then performed using IBM SPSS Statistics, version 29 (IBM Corp., Armonk, NY, USA). Statistical significance was set at p < 0.05. Primary analyses focused on between-group differences in VR2 performance outcomes. Between-group comparisons were performed via unpaired t-tests, whereas paired t-tests were used for within-subject comparisons over time^35,48^. When assumptions of homogeneity of variance were violated, Welch’s t-test was applied^49,50^. Associations between variables were assessed via Pearson correlations. Differences across more than two groups or time points were examined via one-way or mixed ANOVA. Effect sizes were calculated to evaluate the practical relevance of statistically significant results, with Cohen’s *d* reported for t-tests and partial $\eta ^2$ for ANOVA, interpreted according to Cohen^35^.

Baseline characteristics were summarized descriptively by randomized group; no null-hypothesis significance tests were performed.

For the primary comparisons specified in the analysis plan, parametric procedures were retained because the main performance outcomes were treated as approximately continuous measures and group sizes were sufficiently large for robust estimation. Homogeneity of variance was assessed for between-group comparisons, and Welch’s correction was applied where appropriate. Formal normality tests were not used as exclusionary decision rules for selecting statistical procedures. The primary analyses focused on a limited set of VR2 performance outcomes specified in the analysis plan. Secondary questionnaire-based, subgroup, and correlation analyses were interpreted as exploratory. No formal adjustment for multiple comparisons was applied.

Questionnaire outcomes were measured and analyzed at the individual participant level. In contrast, checklist scores and time-based performance outcomes represented the joint performance of each dyad. One set of values was recorded per simulation run and linked to both team members. The reported performance analyses treated participants as the unit of analysis and therefore did not fully account for within-dyad dependence. The implications of this analytical approach for the effective sample size and inferential precision are addressed in the Limitations section.

Missing questionnaire items were excluded from the subscale calculations without imputation. This approach assumes that missingness did not systematically depend on observed outcomes. Participants with incomplete datasets or untraceable pseudonym codes were excluded from the analysis (complete-case analysis). No sensitivity analyses were performed for missing data. No important changes to the trial design or outcome definitions were made after trial commencement. The analysis plan reported in the retrospective OSF registration was used for the present manuscript; analyses not specified there are treated as exploratory.

### Ethical approval

The study program was reviewed and approved by the Ethics Committee of Witten/Herdecke University, Witten, Germany (reference number: S-74/2024). Ethical approval was granted prior to study initiation. All procedures were conducted in accordance with the Declaration of Helsinki as well as relevant national and institutional guidelines and regulations.

Prior to participation, all participants received comprehensive oral and written information regarding the study objectives, procedures, potential risks, and data handling. Written informed consent was obtained from all participants before enrollment. Participation was strictly voluntary, and it was explicitly stated and ensured that study participation or nonparticipation had no influence on the participants’ vocational paramedic training or educational assessment. The participants were free to withdraw from the study at any time without any adverse consequences.

Data were pseudonymized at the point of collection using participant-generated four-character codes. No list linking pseudonym codes to personal identifiers was created or stored. All analyses were conducted without any means of reidentification. No video or audio recordings were created, stored, or published at any stage of the study.

## Results

### Participant flow

Of the 145 paramedic trainees assessed for eligibility, four declined participation before randomization because of prior VR-related discomfort or data protection concerns. Sixteen randomized participants were excluded from the final analysis because of incomplete datasets or untraceable pseudonymized identification codes. Accordingly, a complete-case analysis was performed. All VR simulations were delivered as intended according to the predefined standardized workflow. No participant withdrew after randomization, no predefined stopping criterion was triggered during the trial, and no VR simulation was aborted. The final analytic sample comprised 125 participants (VR-I: n = 64; VR-C: n = 61). The odd number of participants in the final VR-C dataset resulted from participant-level complete-case exclusions after the dyadic simulations; no simulation was conducted by a single participant.

### Baseline characteristics

Table 2 summarizes key demographic, training-related, and VR-related baseline characteristics by randomized group. The groups were descriptively similar with respect to age, gender, year of training, prior emergency medical technician (EMT) qualification, prior VR experience, and corrective-glasses use, with no marked baseline imbalances.

Among participants with available age data, the overall mean age was 24.4 years (SD = 4.4; range 18–46 years). Of the 125 participants, 53.6% were in their first year of training, 68.0% were male, 84.8% had previously completed EMT training, and 36.8% reported prior experience with VR technology.

**Table 2:** Baseline demographic, training-related, and VR-related characteristics by randomized group.

**Characteristic**		**Group**		
		**VR-I**	**VR-C**	**Total**
Total participants, n		64	61	125
Training year, n (%)	First year	32 (50.0)	35 (57.4)	67 (53.6)
	Second year	32 (50.0)	26 (42.6)	58 (46.4)
Gender, n (%)	Male	43 (67.2)	42 (68.9)	85 (68.0)
	Female	21 (32.8)	18 (29.5)	39 (31.2)
	Missing	0 (0.0)	1 (1.6)	1 (0.8)
Age, years, mean (SD)$^1$		24.75 (4.67)	24.03 (4.07)	24.40 (4.40)
Prior EMT qualification$^2$, n (%)		56 (87.5)	50 (82.0)	106 (84.8)
Corrective-glasses use, n (%)		20 (31.3)	24 (39.3)	44 (35.2)
Prior VR experience, n (%)		25 (39.1)	21 (34.4)	46 (36.8)

$^1$Age data were available for 116 of 125 participants; means and standard deviations are based on available data. The overall age range was 18–46 years.

$^2$Prior EMT qualification refers to completed training as a Rettungssanitäter/in in Germany.

### Prior exposure to the identical VR scenario was associated with higher VR2 performance

Performance in the common VR2 assessment scenario differed substantially between groups (see Table 3). Jointly assessed scenario-performance scores were higher in the VR-I group across all domains of the scenario-specific checklist than in the VR-C group. Members of the VR-I dyads had previously completed the identical anaphylaxis scenario, whereas members of the VR-C dyads encountered the scenario for the first time during VR2. The mean total score was 42.6 points in VR-I compared with 31.1 points in VR-C (maximum 62 points; mean difference 11.5 points, 95% CI, 9.8 to 13.3, *t*(123) = 13.4, *p* < 0.001, *d* = 2.39). The most pronounced differences were observed in the first look and BPR anaphylaxis treatment pathway domains, whereas the SSSS schema was rarely performed in either group.

**Table 3:** Performance scores and time-based outcomes of the scenario-specific checklist, stratified by group and VR simulation.

Outcome	VR-I		VR-C	
	Score/Time	%	Score/Time	%
**VR1**$^1$**: Performance scores**				
SSSS	0.02	1.56	—	—
xABCDE	10.47	47.59	—	—
First Look	1.50	50.00	—	—
SAMPLER	4.03	57.59	—	—
BPR	18.27	62.98	—	—
**Total score**	**34.28**	**55.29**	—	—
*Time-based outcomes*$^3$				
Time to correct working diagnosis	3.38 ± 1.80		—	
Time to first IM epinephrine	7.09 ± 2.36		—	
Total scenario duration	14.51 ± 2.46		—	
**VR**2$^2$**: Performance scores**				
SSSS	0.00	0.00	0.03	3.28
xABCDE	12.36	56.18	9.43	42.85
First Look	2.41	80.21	1.33	44.26
SAMPLER	5.48	78.35	4.05	57.85
BPR	22.34	77.05	16.21	55.91
**Total score**	**42.59**	**68.70**	**31.05**	**50.08**
*Time-based outcomes*$^3$				
Time to correct working diagnosis	1.45 ± 0.71		2.90 ± 1.47	
Time to first IM epinephrine	4.58 ± 1.49		7.43 ± 1.68	
Total scenario duration	11.73 ± 1.00		12.94 ± 1.15	

$^1$Only participants in the VR-I group completed VR simulation 1.

$^2$ All participants completed VR simulation 2.

$^3$ Time-based outcomes are presented as the means ± standard deviations (minutes).

**Fig. 4:**
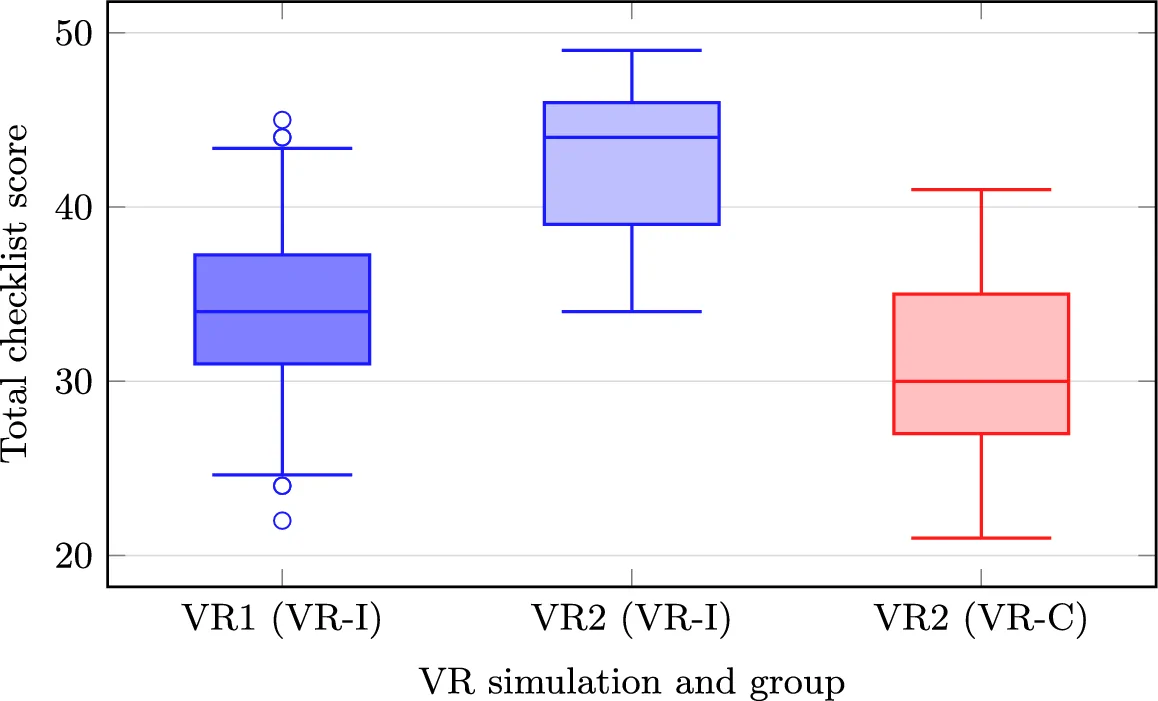
Distribution of total scores across VR simulations and groups. Boxplots display medians, interquartile ranges, whiskers, and outliers.

Consistent with these score differences, the correct working diagnosis was established and IM epinephrine was administered earlier in VR-I than in VR-C. The mean time to correct working diagnosis was 1.45 min shorter in VR-I than in VR-C (1.45 vs. 2.90 min; mean difference, −1.45 min; 95% CI, −1.86 to −1.03; *t*(85.53) = −6.9, *p* < 0.001, *d* = 1.15; Welch’s t-test). The mean time to first IM epinephrine administration was 2.85 min shorter in VR-I than in VR-C (4.58 vs. 7.43 min; mean difference, −2.85 min; 95% CI, −3.41 to −2.24; *t*(115) = −9.58, *p* < 0.001, *d* = 1.59; Welch’s t-test). The checklist criterion for IM epinephrine administration was fulfilled in 100% of participant-linked VR-I observations and 87% of participant-linked VR-C observations during VR2. Fig. 4 summarizes the distribution of total scores across groups and VR simulations.

Descriptively, first-exposure performance was broadly similar across groups: the VR-I group achieved 34.28 points during VR1, whereas the VR-C group achieved 31.05 points during VR2, which was their first exposure to the scenario. By contrast, the VR-I group achieved 42.59 points during VR2 after repeated exposure to the identical scenario. This pattern supports the interpretation that the large VR2 between-group difference primarily reflects repeated exposure and scenario familiarity rather than a generalized baseline difference between groups.

**Fig. 5:**
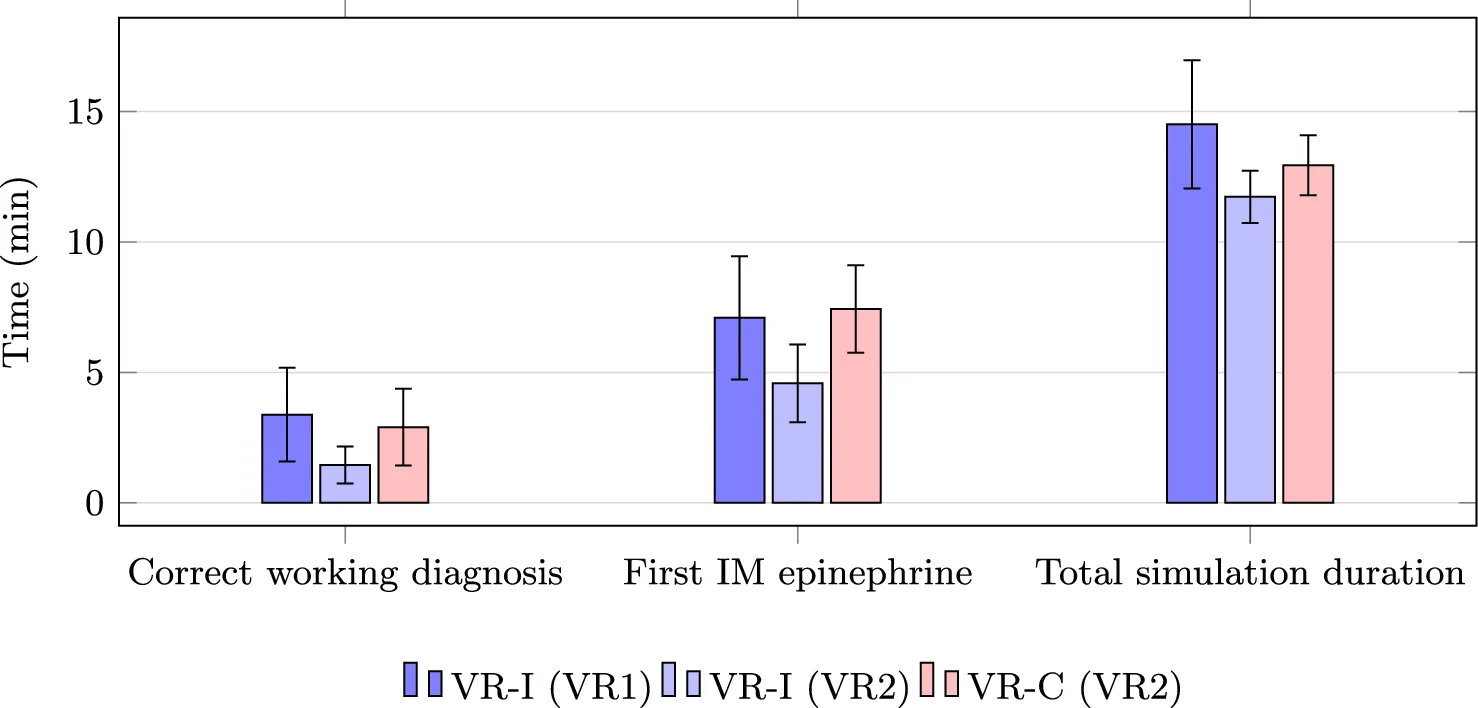
Mean time intervals (± SD) across VR simulations and groups.

Both time-based outcomes correlated strongly and negatively with overall performance (diagnosis: *r* = −0.593, *p* < 0.001; IM epinephrine administration: *r* = −0.711, *p* < 0.001). Figure 5 summarizes the time-based outcomes across groups and VR simulations.

### Secondary outcomes

#### No corresponding between-group differences in knowledge test scores

**Fig. 6:**
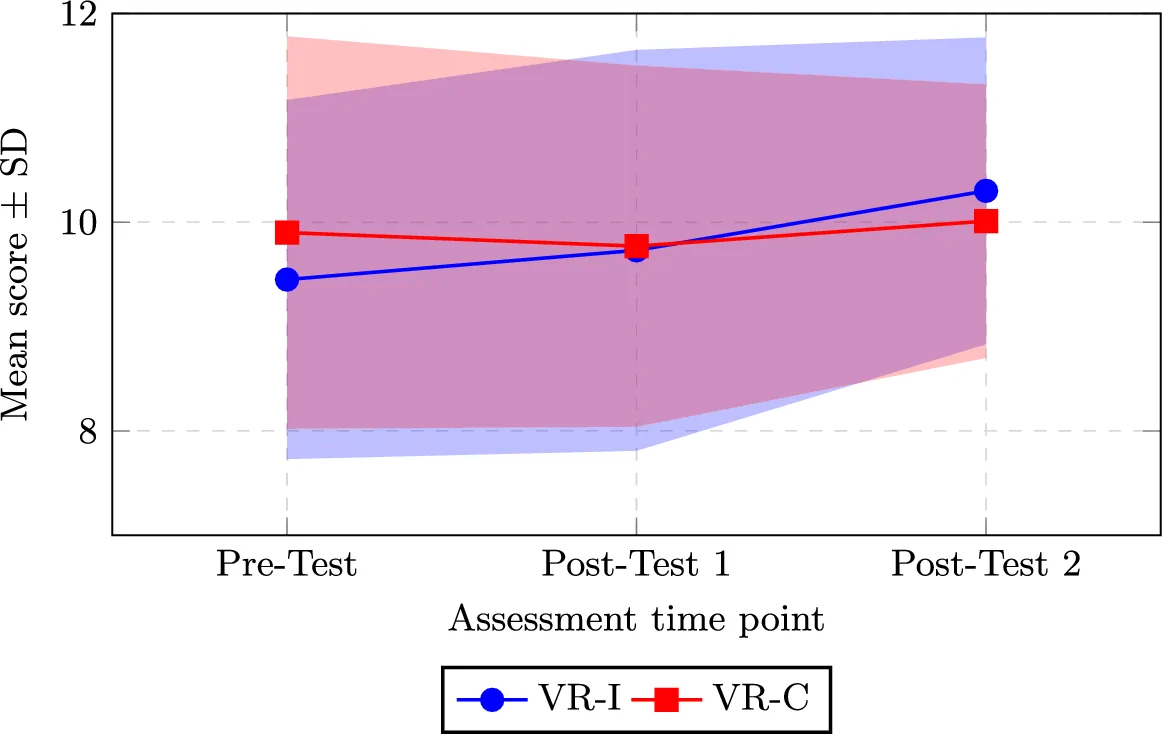
Mean medical knowledge scores for the VR-I and VR-C groups across pre-test, post-test 1, and post-test 2. The shaded areas represent ± SD. Maximum achievable score: 14 points.

The performance of knowledge tests on anaphylaxis management was largely comparable across groups at all assessment points (see Fig. 6 and Table 4). While scores in the VR-I group showed a modest increase over time, mixed ANOVA revealed a significant effect of time (*F*(2, 246) = 5.09, *p* = 0.005, partial $\eta ^2$ = 0.04) but no significant effect of group (*F*(1, 123) = 0.08, *p* = 0.775) or time *×* group interaction (*F*(2, 246) = 2.67, *p* = 0.072, partial $\eta ^2$ = 0.021). In line with the secondary objective of examining coherence between declarative knowledge and objectively assessed scenario performance, knowledge test scores did not correlate significantly with evaluation scores.

Thus, the large between-group difference in VR2 performance was not mirrored by a corresponding between-group difference in declarative knowledge scores. This discrepancy suggests that repeated exposure may primarily have enhanced scenario-specific recognition, procedural sequencing, and execution within the VR environment rather than producing a clearly measurable gain in written anaphylaxis knowledge.

**Table 4:** Medical knowledge scores on guideline-based anaphylaxis management by group and assessment time point (mean ± SD).

Assessment time point	Group	
	VR-I	VR-C
Pre-Test	9.45 ± 1.72	9.90 ± 1.88
Post-Test 1	9.73 ± 1.92	9.77 ± 1.73
Post-Test 2	10.30 ± 1.47	10.01 ± 1.31

Maximum achievable score: 14 points.

#### VR performance was not significantly associated with assessed individual factors

No significant associations were found between VR simulation performance and prior VR experience, age, gender, or professional background.

#### VRISE symptoms were only weakly associated with assessed individual factors

The participants reported generally low to moderate levels of VRISE during and after the VR simulations. Symptom intensity varied between individuals, but no participant withdrew from the study, and no simulation was terminated owing to adverse effects. VRISE scores showed a weak positive correlation with participant age (*r* = 0.221, *p* = 0.015) and a weak negative correlation with corrective-glasses use (*r* = −0.183, *p* = 0.044).

**Table 5:** Descriptive statistics for the five VR experience subscales and the single-item presimulation topic-awareness measure derived from the 18-item VR questionnaire.

	Group	
	VR-I	VR-C
**VR1**$^1$		
Immersion	2.65 ± 1.00	—
Competency progression	2.73 ± 0.88	—
Usability	2.17 ± 0.94	—
Attitudes toward VR Integration	2.17 ± 0.88	—
VRISE	1.18 ± 1.22	—
Pre-simulation Topic Awareness$^2$	—	—
**VR2**$^3$		
Immersion	2.43 ± 1.10	2.40 ± 1.01
Competency progression	2.56 ± 1.05	2.35 ± 1.01
Usability	2.15 ± 1.09	1.78 ± 1.16
Attitudes toward VR Integration	1.91 ± 1.07	1.77 ± 1.16
VRISE	1.27 ± 1.29	1.69 ± 1.36
Pre-simulation Topic Awareness	2.25 ± 1.44	1.42 ± 1.46

Items were scored from 0 (*strongly disagree*) to 4 (*strongly agree*). Higher scores indicate stronger agreement with the respective statement; for the VRISE subscale, higher scores indicate greater symptom burden. Multi-item subscale scores were calculated as item means. Standard deviations were approximated from item-level variability and are reported for descriptive purposes.

$^1$Only participants in the VR-I group completed VR simulation 1.

$^2$ No data are reported for item 18 in the VR-I group at VR1 simulation (post-test 1), because prior awareness of the scenario was not expected at this time point.

$^3$ All participants completed VR simulation 2.

No significant correlations were found with gender, total simulation duration, frequency of VR exposure, or prior VR experience. Detailed descriptive statistics are presented in Table 5.

#### Reflections on using VR in the paramedic context

The participants generally regarded VR as a valuable adjunct to conventional paramedic training rather than a replacement. Across simulations, immersion and acceptance were rated positively, and participants perceived the scenarios as realistic and engaging. Notably, the VR-I group consistently reported greater preparedness and a greater ability to apply existing professional competencies than did the VR-C group.

Perceived educational value was also greater in the VR-I group and correlated weakly but significantly with objective performance scores (*r* = 0.277, *p* < 0.001). Technical aspects, including graphic quality and communication between participants, were generally rated positively, although some variability in system latency was noted. Overall, the participants did not perceive VR scenarios as faster than real-life emergency responses.

Participants reported that insights from previous curricular full-scale simulation debriefings on anaphylaxis, conducted outside the VR intervention, were helpful for navigating the VR scenarios. The anticipation of anaphylaxis was higher in the VR-I group and correlated weakly but significantly with performance in the second VR simulation (*r* = 0.254, *p* < 0.001). Detailed descriptive statistics for all the subjective measures, including preparedness, immersion, and perceived educational benefits, are presented in Table 5.

## Discussion

This randomized controlled trial showed that repeated exposure to an identical, standardized multi-user VR anaphylaxis scenario was associated with improved subsequent performance within the same VR-based emergency scenario. The VR-I group showed higher jointly assessed guideline-based performance scores, and the correct working diagnosis and first IM epinephrine administration occurred substantially earlier than in the VR-C group.

The findings should therefore be interpreted as evidence of improved scenario-specific performance after repeated VR exposure, not as evidence of transfer to different emergency conditions, conventional simulation settings, or real-life paramedic performance. This distinction is central to the interpretation of the trial. The intervention did not compare VR with another educational modality. Rather, it tested whether an additional exposure within a standardized VR environment improved performance during a later exposure to the same scenario. Similar repetition effects may also occur in conventional simulation settings. The present trial does not establish that these mechanisms are unique to VR.

The magnitude of the observed effects is educationally plausible in this context. The scenario followed a highly standardized, guideline-driven anaphylaxis workflow with visually salient cues, including urticaria and upper airway involvement, and a clearly defined treatment pathway. Repeated exposure may therefore have facilitated faster pattern recognition, greater familiarity with the virtual environment and equipment layout, more fluent procedural sequencing, and earlier execution of expected time-critical actions. These mechanisms are consistent with deliberate rehearsal in a reproducible simulation environment, but they do not by themselves demonstrate broader clinical competence.

Importantly, no structured debriefing or corrective teaching was provided between VR1 and VR2. The observed differences therefore cannot be attributed to formal instructor feedback after VR1. However, prior exposure itself may have increased familiarity with the scenario, reduced cognitive load, and increased anticipation of anaphylaxis, all of which may have contributed to the substantial between-group separation observed in VR2. Future studies should examine whether integrating structured debriefing after VR exposure further enhances learning, retention, and transfer to unfamiliar scenarios or non-virtual performance settings.

Participants also reported drawing on insights from curricular full-scale anaphylaxis simulation debriefings conducted outside the allocated VR intervention. The timing and individual extent of this prior curricular exposure were not systematically documented, and its independent contribution therefore cannot be isolated. Because randomization was performed within training cohorts, such background exposure would be expected to have been broadly distributed across groups, although residual imbalance cannot be excluded.

In addition to performance, the present study contributes to the ongoing discussion regarding the feasibility and safety of immersive VR training in emergency care education^5,16,30,32^. The VRISE scores were generally mild to moderate and did not lead to discontinuation of any VR simulation. In line with prior evidence suggesting potential age-related susceptibility to VRISE^30^, we observed a weak but significant association between increased age and symptom reporting. Conversely, gender was not associated with VRISE, corroborating previous findings indicating no consistent gender effect^30^. Although previous research has proposed longer exposure durations and habituation effects across repeated VR sessions as relevant moderators of VRISE^29^, neither total simulation duration nor prior VR exposure showed a significant association with symptom burden in the present study. Notably, wearing corrective glasses was associated with slightly lower VRISE scores. This factor has received little attention in the literature and may represent a previously underexplored individual characteristic with potential relevance for future screening or risk stratification in educational VR settings. Overall, these findings suggest that the VR simulations were feasible and well tolerated within this preselected trainee sample. However, the tolerability findings should not be generalized without caution to less preselected learner populations, particularly individuals with prior VR-related discomfort.

While the present study focused on performance within VR simulations, it also informs the broader question of usability and acceptability of VR-based training in EMS schools. The observed performance gains within the VR environment and the favorable tolerability profile support the use of VR as a feasible adjunct for repeated rehearsal of standardized, time-critical emergency workflows. They do not, however, establish transfer to real-life emergency care or superiority over conventional simulation. This complements prior reports of positive learner attitudes and user experience in VR-based teaching and assessment^16,51–53^.

The absence of corresponding between-group differences in written knowledge scores is a key interpretive finding. It suggests that the intervention did not primarily increase declarative anaphylaxis knowledge as measured by the questionnaire. Instead, repeated exposure appears to have improved the application of existing knowledge within a familiar, cue-rich, time-critical VR scenario. In educational terms, the intervention may have strengthened recognition, prioritization, procedural fluency, and execution under simulated pressure rather than broad conceptual knowledge acquisition. This interpretation is consistent with the procedural and timing-sensitive structure of the assessment instrument.

Recent randomized evidence from emergency healthcare training suggests that immersive technologies can improve post-intervention knowledge compared with traditional instruction, with VR showing the largest gains, whereas satisfaction differences may be less robust^54^. In contrast to these primarily knowledge-based endpoints, our trial extends the evidence base by examining scenario-specific performance and time-critical decision-making within a standardized VR assessment context (e.g., time to correct working diagnosis and IM epinephrine). This distinction is relevant for paramedic education, where the ability to execute guideline-based care rapidly and reliably under pressure constitutes a core training objective.

Our results align with earlier work reporting positive educational effects of virtual simulation-based training via the same software and a comparable clinical scenario (grade III anaphylaxis)^16^. Similar to the study by Lerner et al.^16^, no VR simulations had to be discontinued due to VRISE. Importantly, the present study extends these initial observations by reporting substantial scenario-specific performance differences in a larger and more systematically assessed sample.

In parallel with continued advances in VR technology^15,55,56^, VR-based training is likely to become increasingly accessible and scalable for EMS education. However, the effective educational integration of VR requires more than hardware or software alone. As prior evidence suggests, the availability of digital simulation technologies in itself does not suffice to modernize and improve education^21,57,58^. Instead, successful implementation in paramedic schools should be accompanied by deliberate pedagogical adaptation and systematic training of teachers and instructors.

Previous comparative studies have reported potential benefits of VR-based training for learner engagement, self-efficacy, and training efficiency in selected emergency education contexts^53^. These findings should, however, be distinguished from the present trial, which did not compare VR with conventional simulation or other instructional formats. In our study, the educational contribution of VR lies primarily in its ability to provide a reproducible, multi-user environment for repeated rehearsal of the same time-critical anaphylaxis workflow. From an implementation perspective, VR requires initial technical infrastructure and scenario development. Once implemented, however, digitally predefined scenarios can be repeated under highly standardized conditions, which may facilitate reproducible practice and assessment with less variability than many analog simulation formats. Collectively, the available evidence suggests that VR may complement emergency medical education when embedded in a deliberate pedagogical design, but its relative effectiveness compared with established simulation modalities remains an empirical question for future research.

Recent comparative work in paramedic education further supports the need for deliberate pedagogical integration of immersive technologies. In particular, mixed reality (MR) and VR interventions appear to have full educational potential only when they are intentionally planned and guided by pedagogical professionals rather than deployed as stand-alone technological add-ons^52^. From a didactic standpoint, instructors play a central role not only in structuring learning objectives and scenario flow but also in explicitly addressing and contextualizing the inherently artificial aspects of virtual simulations. Facilitating learners’ acceptance of these otherwise unrealistic elements has been identified as a prerequisite for meaningful engagement and effective learning within immersive environments^52^. This highlights that pedagogical responsibility remains paramount in VR- and MR-based training, even as technological realism continues to improve.

A key challenge will be to translate established, evidence-based instructional approaches from conventional paramedic education into virtual environments while preserving their pedagogical effectiveness. Addressing this challenge may allow hybrid curricula to combine the strengths of analog and digital simulation in a manner that is both educationally robust and sustainable at scale.

## Limitations

Several limitations should be considered when interpreting these findings. First, this was a single-center trial conducted at one German paramedic training academy. Generalizability to other EMS schools, curricula, instructor teams, VR systems, and learner populations may therefore be limited.

Second, the study assessed repeated exposure using a single anaphylaxis scenario and did not include an active comparator or retention assessment. Consequently, the findings do not establish longer-term retention, transfer to unfamiliar scenarios or non-virtual settings, or superiority over conventional simulation. No additional study-provided VR practice, debriefing, or anaphylaxis simulation occurred between exposures; however, routine curricular teaching and real-world clinical exposure outside the study were not systematically recorded.

Third, the scenario-specific checklist was developed for this study and underwent informal expert content review by two physicians with prehospital emergency medicine qualifications, but it was not subjected to formal psychometric validation, pilot testing, or reliability testing. Performance was assessed in real time by a single, unblinded study coordinator, and inter-rater reliability could therefore not be determined. In addition, clinically central actions were represented by multiple checklist criteria, which may have increased the contribution of rapid recognition and execution of the expected treatment pathway to the total score.

Fourth, participants were individually randomized, whereas scenario performance was generated jointly by dyads. One checklist score and one set of time-based outcomes were recorded per simulation run and linked to both team members. The participant-level analyses therefore did not fully account for within-dyad dependence, which may have overestimated the effective sample size and the precision of confidence intervals and p values. The direction and magnitude of the observed differences remain informative, but the exact inferential estimates should be interpreted cautiously. Because dyad composition could change between VR1 and VR2 and could not be reliably reconstructed for every simulation, a post hoc longitudinal dyad-level analysis was not feasible. Associations between individual participant characteristics and shared dyad-level performance outcomes should likewise be regarded as exploratory.

Fifth, the VRISE findings are affected by preselection. Trainees who did not feel physically capable of participating or who declined participation because of prior VR-related discomfort were not randomized. The observed frequency and intensity of VRISE may therefore underestimate symptom burden in less preselected learner populations.

Finally, blinding was not feasible because of the nature of the intervention. Participants and the study coordinator were aware of group allocation, and performance and assessment bias cannot be excluded. Secondary questionnaire-based, subgroup, and correlation analyses were exploratory and were not adjusted for multiple comparisons.

## Conclusions

Prior exposure to the same anaphylaxis scenario was associated with substantial improvements in subsequent scenario-specific performance among paramedic trainees. The VR-I group showed higher jointly assessed guideline-based checklist scores and shorter times to correct working diagnosis and first IM epinephrine administration than the VR-C group.

VR-based training was well tolerated in this preselected trainee sample, with generally mild VRISE and no simulation discontinuations. These findings support immersive VR as a reproducible environment for deliberate rehearsal of time-critical emergency workflows. However, the results should be interpreted as evidence of improved performance within a familiar VR-based procedural context, not as proof of generalized clinical competence, real-world transfer, or superiority over conventional simulation. Future research should evaluate multicase designs, active comparator conditions, longer-term retention, and transfer to unfamiliar scenarios and non-virtual performance settings.

## Supplementary Information

Supplementary Information.

## Data Availability

The datasets used and/or analyzed during the current study are available from the corresponding author upon reasonable request.

## Abbreviations

BPR: Behandlungspfad Rettungsdienst (German paramedic treatment pathway)
EMS: Emergency Medical Services
EMT: Emergency Medical Technician
HMD: Head-mounted display
IM: Intramuscular
MR: Mixed Reality
SAMPLER: Symptoms, allergies, medications, past medical history, last oral intake, events leading to the incident, and risk factors
SD: Standard deviation
SiNA: Simulation and Emergency Academy at Helios Hospital Krefeld, Germany
SSSS: Scene, safety, support, and situation
VR: Virtual Reality
VR-C: Virtual Reality control group
VR-I: Virtual Reality intervention group
VRISE: Virtual Reality-Induced Symptoms and Effects
xABCDE: Exsanguination, airway, breathing, circulation, disability, and exposure

## Materials availability

Additional study materials may be made available by the corresponding author upon reasonable request.
