# Supplementary material for: Effect of repeated exposure to an identical multi-user virtual reality anaphylaxis scenario on paramedic trainee performance: a randomized controlled trial
Source: Sci Rep. 2026 Sep 3;16:27651. doi: 10.1038/s41598-026-69133-x (PMC13542146; doi:10.1038/s41598-026-69133-x)
Supplement: Supplementary file 1 [file 41598_2026_69133_MOESM1_ESM.pdf]

9 Supplementary Information

The supplementary information provides the full study instruments and questionnaires used in this study, including all the items, response formats, and scoring procedures.

Supplementary Information: Standardized workflow and management of the VR anaphylaxis scenario

Table 6: Standardized workflow and management of the VR anaphylaxis scenario

**Framework conditions**		
Time of emergency	3:30 PM	
Season	Summer	
Dispatch keyword	"Unclear internal condition" (one ambulance dispatched)	
Location	Residential setting in a garden, single-family neighborhood	
Crew composition	1 paramedic, 1 emergency medical technician	
Nearby hospitals	Primary care hospital: approx. 10 minutes driving time<br>Tertiary care hospital: approx. 20 minutes driving time	
**Situation – leading symptoms – injury pattern – diagnosis**		
The ambulance crew is met on the street by a neighbor and guided to the terrace. The patient is seated, reporting marked shortness of breath. The neighbor reports that while having coffee and cake, the patient suddenly felt unwell despite being previously healthy and requested that emergency services be called. Weight, age, and body habitus are as predefined by the simulation scenario.		
**General impression/first look**		
Respiration	Patient responsive, speech limited to short sentences	
Circulation	Peripheral pulse weakly palpable, tachycardia	
Neurology	Delayed response, muscle tone preserved, eyes open	
**Medical history – patient information – SAMPLER**		
Symptoms	Sudden shortness of breath, generalized itching, pronounced anxiety	
Allergies	Nuts (no further details provided by patient)	
Medications	No chronic medication	
Past medical history	Only known nut allergy with a single, mild anaphylactic reaction several years ago	
Last meal	approx. 20–30 minutes ago, a piece of cake<br>(baked and delivered by a neighbor)	
Event	Cake consumption, previously in good health	
Risk factors	None	
**Vital signs**	**Primary Survey**	**Secondary Survey**
**x**	No visible critical external bleeding	No visible critical external bleeding

Continued on next page

Table 6 – Continued from previous page

**A**irway	Marked tongue and uvula swelling, no visible secretions, inspiratory stridor audible	Tongue and uvula swelling decreasing but persistent, no visible secretions, inspiratory stridor decreasing
**B**reathing	Respiratory rate: 30/min<br>SpO_2: 88% on FiO_2 0.21<br>Auscultation: bilateral inspiratory stridor	Respiratory rate: 23/min<br>SpO_2: 94% on $FiO_2 \geq 0.60$<br>Auscultation: bilateral vesicular breath sounds, mild inspiratory stridor present
**C**irculation	Pulse: weakly palpable peripherally, bilateral<br>Capillary refill time: approx. 3 seconds, bilateral<br>Heart rate: 140/min<br>Blood pressure: 75/40 mmHg<br>ECG: sinus tachycardia	Pulse: weakly palpable peripherally, bilateral<br>Capillary refill time: < 2 seconds<br>Heart rate: 120/min<br>Blood pressure: 110/75 mmHg<br>ECG: sinus tachycardia
**D**isability	GCS: 15<br>Pupils: isocoric, prompt light reaction, mid-dilated<br>Blood glucose: 130 mg/dL<br>Mental status: anxious, restless	GCS: 15<br>Pupils: isocoric, prompt light reaction, mid-dilated<br>Blood glucose: 130 mg/dL<br>Mental status: cooperative, calm
**E**xposure/ Environment	Body temperature: 37.0°C<br>Skin: erythematous, urticaria<br>No accompanying injuries observed	Body temperature: 37.0°C<br>Skin: blanching, mildly erythematous, urticaria decreasing<br>No accompanying injuries observed

Supplementary Questionnaire B: Standardized Scenario-specific Performance Assessment Checklist

Table 7: Standardized Scenario-specific Performance Assessment Checklist

**Scene, safety, support, and situation**	
O	The scene was assessed orientationally, safety confirmed, and the need for immediate additional resources was negated.
**General Impression/First Look**	
O	An initial orienting assessment of the patient was performed.
O	The critical condition was recognized.

Continued on next page

Table 7 – Continued from previous page

O	The call-out "critical patient" was communicated to team members.
**xABCDE**	
O	x – The presence of an acute, critical external bleeding was excluded.
O	A – The oral cavity was inspected.
O	A – Tongue/uvula swelling was detected (identified as a potential airway problem).
O	B – Respiratory rate was counted.
O	B – Respiratory rate was assessed as pathological tachypnea.
O	B – SpO_2 was measured.
O	• The measured SpO_2 was interpreted as pathological/hypoxic.
O	B – Bilateral lung auscultation was performed.
O	• Inspiratory stridor was detected.
O	B – An orienting inspection for possible B-problems, e.g., symmetrical thoracic excursions or distended neck veins, was performed.
O	C – The peripheral pulse was palpated.
O	• Estimated/measured pulse rate and rhythm were evaluated as pathological.
O	C – At least a 4-lead ECG monitoring was used.
O	C – Arterial blood pressure was measured at least once.
O	D – Blood glucose was measured.
O	D – Pupillary reaction was tested.
O	D – Consciousness was assessed orientationally (AVPU scale).
O	D – GCS was assessed.
O	E – Body temperature was measured.
O	E – Clothing was sufficiently removed for further diagnostics and identification of potential E-problems.
O	E – Skin conditions were assessed, and urticaria was detected.
O	xABCDE – A-, B-, and C-problems were identified.
**SAMPLER**	
O	All relevant symptoms were assessed.
O	All relevant allergies were assessed.
O	All relevant medications/prior medications were assessed.
O	All relevant points of the patient's medical history were assessed.
O	All relevant points of the last meal were assessed.
O	All relevant points of the triggering event were assessed.
O	All relevant risk factors were assessed.
**BPR "Anaphylaxis"**	
O	Correct working diagnosis "anaphylaxis" was established.
O	On the basis of clinical findings, anaphylactic shock was recognized/reported as grade III.
O	Additional emergency physician dispatch was requested.
O	The predominant airway problem was identified.
O	Oxygen therapy was administered.

Continued on next page

Table 7 – Continued from previous page

O	• 10–15 L/min oxygen via face mask or oxygen via nebulizer mask was given.
O	Intramuscular epinephrine administration was performed.
O	• Into the mediolateral third of the thigh (M. vastus lateralis).
O	• Using undiluted epinephrine.
O	• Dose: 0.5 mg epinephrine.
O	Inhaled epinephrine administration was performed.
O	• Dose: 2.0 mg epinephrine.
O	• Together with 2.0 mL of 0.9% sodium chloride in the nebulizer chamber.
O	At least one peripheral intravenous access was established.
O	• With administration of a full-electrolyte solution as part of targeted, generous volume resuscitation (500–1,000 mL intravenous).
O	Intravenous prednisolone administration was performed.
O	• Dose: 250 mg prednisolone.
O	Intravenous dimetindene administration was performed.
O	• Dose: 4–8 mg dimetindene (0.1 mg/kg body weight, max. 8 mg).
O	The patient was cared for through appropriate communication.
O	Patient positioning was symptom-oriented (most likely sitting).
O	At least one structured team time-out (e.g., 10-for-10) was conducted.
O	• The xABCDE scheme was fully repeated as part of a secondary survey.
O	• Clinical symptom improvement was identified.
O	A body check (for further injuries, possible preventable exposures) was conducted.
O	Transport was initiated/prepared.
O	Continuous monitoring of patient and vital signs was performed.
O	Abnormalities in vital status were communicated to team members (e.g., during xABCDE).
O	The patient's condition was continuously reevaluated (at least twice during the course, or at least every 5 minutes, or with every change in situation).
**Times**	
	Time to establishment of correct working diagnosis (min): ___ Time to first intramuscular epinephrine administration (min): ___ Total duration of this simulation (min): ___

Supplementary Questionnaire C: Sociodemographic and education-related data

Table 8: Sociodemographic and education-related data

**1. How old are you?**	
	___ years
**2. Which gender do you identify with?**	

Continued on next page

Table 8 – Continued from previous page

O	Male
O	Female
O	Diverse / Nonbinary
**3. What is your highest school-leaving qualification?**	
O	Lower secondary school certificate (Hauptschulabschluss)
O	Lower secondary school certificate, grade 10 (Hauptschulabschluss Klasse 10)
O	Intermediate school-leaving certificate (Mittlere Reife/Fachoberschulreife)
O	Advanced technical college entrance qualification (Fachabitur/Fachhochschulreife)
O	General higher education entrance qualification (Abitur)
O	Don't know / no answer
**4. Which professional prequalifications do you have? (multiple answers possible)**	
O	Emergency medical technician (Rettungssanitäter/in)
O	Emergency medical assistant (Rettungshelfer/in)
O	Medical assistant (Sanitätshelfer/in)
O	Other prehospital emergency training
O	Medical administrative assistant (MFA)
O	Medical-technical assistant (MTA)
O	Nursing training
O	Completed nonmedical prequalification
O	No professional prequalification / no answer
**5. In which year did you start your work in emergency services?**	
	In the year ___
**6. Do you wear glasses?**	
O	Yes
O	No
**7. As part of this study, an introduction to VR technology was already provided at SiNA. Did you have any prior experience with VR headsets and/or VR before this introduction?**	
O	Yes
O	No
**8. Are you completing the paramedic training full-time?**	
O	Yes
O	No
**9. In which year of training are you currently?**	
O	1st year of training
O	2nd year of training
O	3rd year of training

Supplementary Questionnaire D: VR simulation and experience

Table 9: VR simulation and experience

No.	Statement	0	1	2	3	4
Response scale: 0 = Strongly disagree; 1 = Somewhat disagree; 2 = Partly agree/partly disagree; 3 = Somewhat agree; 4 = Strongly agree.						
1	The VR simulation felt like a real emergency scenario to me.					
2	I felt deeply immersed in the VR environment.					
3	I could easily detach from external distractions during the VR simulation.					
4	I was able to apply my previously acquired professional skills effectively in the VR simulation.					
5	The VR simulation helped me improve my performance.					
6	I prefer VR simulations over other real-world simulation methods (e.g., full-scale simulations, complex simulations).					
7	I experienced discomfort or unease during the VR simulation (e.g., symptoms such as fatigue, headache, nausea, dizziness, double vision, or difficulty focusing).					
8	I experienced discomfort or unease after the VR simulation (e.g., symptoms such as fatigue, headache, nausea, dizziness, double vision, or difficulty focusing).					
9	I feel that my performance in the VR simulation was faster than in real-life scenarios.					
10	I believe VR simulation should be used more frequently in training.					
11	I felt well prepared for the VR simulation.					
12	Operating the VR headset and controllers was easy for me.					
13	The graphical quality of the VR simulation was high.					
14	Interactions in the VR environment were executed without noticeable delays or stuttering.					
15	I was able to communicate effectively with my teammate during the VR simulation.					
16	I was able to apply insights from the debriefing after the full-scale simulation on "Anaphylaxis" effectively in the VR simulation.					
17	Insights from the debriefing after the full-scale simulation on "Anaphylaxis" helped me perform better in the VR simulation.					
18	I knew before the VR simulation that the scenario would involve anaphylaxis.					

Supplementary Questionnaire E: Medical knowledge on guideline-based anaphylaxis management

Table 10: Medical knowledge on guideline-based anaphylaxis management

**1. According to anaphylaxis registry data, what is the most common trigger of severe anaphylactic reactions in adults?** (Type A+)	
–	Food
√	Insect venom
–	Medications
–	Pollen
–	Latex
**2. Which statement(s) regarding vasoactive substances in the pharmacological management of anaphylaxis is/are correct?** (Type k-prime)	
√	If ineffective, and depending on adverse effects, intramuscular epinephrine can be repeated every five to ten minutes according to clinical symptoms.
–	Intravenous administration of antihistamines (H_1-receptor antagonists) should always precede intramuscular epinephrine.
–	Due to severe cardiac side effects, intramuscular epinephrine is indicated only for patients with pronounced anaphylactic shock.
√	In severe, life-threatening anaphylaxis, there is no absolute contraindication for epinephrine.
√	In addition to intramuscular administration, inhaled epinephrine is particularly effective for treating laryngeal edema.
**3. What is the most important pharmacological agent in the acute treatment of anaphylaxis?** (Type A+)	
–	Ondansetron
–	Norepinephrine
–	Prednisolone
√	Epinephrine
–	Salbutamol
**4. How should anaphylaxis with the leading symptom of bronchial obstruction be treated in addition to intramuscular epinephrine?** (Type A+)	
–	Noninvasive ventilation (NIV)
–	Nebulized 0.9% sodium chloride
–	Sublingual glyceryl trinitrate
–	Prednisolone nebulization
√	Salbutamol nebulization
**5. What intramuscular epinephrine dose should be used for a 48-year-old adult weighing 78 kg?** (Type A+)	
–	0.05 mg epinephrine
–	0.3 mg epinephrine
√	0.5 mg epinephrine

Continued on next page

Table 10 – Continued from previous page

–	2 mg epinephrine
–	5 mg epinephrine
**6. What is the most important treatment principle to minimize complications of anaphylactic shock?** (Type A+)	
–	Rapid transport to an appropriate target hospital ("load and go")
–	Emergency anesthesia
–	Early, calculated intravenous broad-spectrum antibiotics
–	High-flow oxygen therapy
√	Rapid catecholamine therapy
**7. Which statement regarding allergies and anaphylactic reactions is false?** (Type A-)	
√	Owing to rapid drug absorption, intramuscular administration in the deltoid muscle is preferred.
–	The tissue hormone histamine plays a key role in anaphylactic reactions.
–	Type I allergy is an IgE-mediated immediate hypersensitivity reaction.
–	The most life-threatening symptom of anaphylaxis should be treated first.
–	An anaphylactic reaction can stop spontaneously at any severity level and regress over time.
**8. Which of the following diagnoses does not represent a relevant differential diagnosis for anaphylaxis?** (Type A-)	
–	Cardiogenic shock
√	Primary tuberculosis
–	Alcohol intoxication
–	Pulmonary embolism
–	Tracheal/bronchial obstruction (e.g., foreign body)
**9. Which algorithm allows life-threatening conditions to be recognized most quickly?** (Type A+)	
–	SAMPLER
–	IPPAF
–	Secondary Survey
–	OPQRST
√	xABCDE
**10. Which of the following statements best defines anaphylaxis?** (Type A+)	
–	Anaphylaxis always represents an allergic reaction to an insect sting.
√	Anaphylaxis is a severe, life-threatening, generalized or systemic hypersensitivity reaction.
–	Anaphylaxis causes a rapid drop in blood pressure, primarily due to direct CNS inhibition without significant immune involvement.
–	Anaphylaxis is a specific form of asthma triggered exclusively by environmental allergens such as pollen or dust.
–	Pathophysiologically, autoantibodies against self-cells are formed, similar to autoimmune diseases.

Continued on next page

Table 10 – Continued from previous page

**11. Which of the following statements about clinical symptoms of anaphylactic reactions is/are correct?** (Type k-prime)	
√	Symptoms of an anaphylactic reaction may occur simultaneously or sequentially in different ways.
–	Cutaneous reactions always precede pulmonary and cardiovascular reactions.
√	Circulatory reactions may occur primarily, without preceding cutaneous or pulmonary symptoms.
√	In up to 20% of cases, the recurrence of symptoms may occur after initially successful treatment.
**12. Which of the following statements about the manifestation of anaphylaxis on the skin and mucous membranes is/are correct?** (Type k-prime)	
–	Itching, redness, urticaria, and angioedema occur only on skin areas directly exposed to the trigger (e.g., an insect sting).
√	Skin is frequently affected in anaphylaxis.
√	Early prodromal symptoms may include itching or burning on the palms and soles.
√	Swelling of the tongue, hypopharynx, or larynx can rapidly become life-threatening.
**13. Which organ systems can be affected by anaphylactic reactions?** (Type k-prime)	
√	Skin
√	Airways
√	Gastrointestinal tract
√	Cardiovascular system
**14. You arrive at an emergency patient. How do you generally proceed with the primary assessment? Arrange the following steps in the correct order (numbers 1–5).** (Type k-prime with specific sequence)	
2	Check: Cyanosis, tachypnea, SpO_2 drop?
5	Check: Skin changes?
1	Check: Airway swelling, hoarseness, stridor?
4	Check: Confusion, unconsciousness?
3	Check: Pallor, cold sweat, hypotension, tachycardia, prolonged capillary refill time?
